# The Gut–Lung–Joint Axis: A Conceptual Framework for Microbiome-Mediated Crosstalk Between Respiratory and Rheumatic Diseases in Childhood

**DOI:** 10.3390/ijms27198692

**Published:** 2026-09-29

**Authors:** Lisa Gazzolari, Laura Di Domenico, Natalie Leone, Saverio La Bella, Paola Di Filippo, Dorina Hoxha, Sabrina Di Pillo, Francesco Chiarelli, Luciana Breda, Daniele Russo, Marina Attanasi

**Affiliations:** 1 Department of Paediatrics, University of Chieti-Pescara, 66100 Chieti, Italy; 2 School of Medicine and Surgery, University of Milano-Bicocca, 20126 Milan, Italy

**Keywords:** gut–lung–joint axis, gut microbiome, microbiota, paediatric asthma, juvenile idiopathic arthritis, dysbiosis, immune regulation, PubMed

## Abstract

There is growing interest in the role of the gut microbiome in immune development and homeostasis during childhood. Increasing evidence suggests that early alterations in microbial composition might influence susceptibility to both respiratory and rheumatic diseases through interconnected pathways. While the gut–lung and gut–joint axes have been investigated separately, their integration into a biologically specific and testable model remains largely unexplored. In this narrative review, the literature was searched in PubMed/MEDLINE and Scopus for evidence syntheses published from 2020 to 2026; 37 eligible evidence syntheses were included, comprising 26 addressing the gut–lung/respiratory domain and 11 addressing the gut–joint/rheumatic domain. We examine current knowledge of microbiome-mediated interactions linking the gut, lungs, and joints in childhood. We discuss the development of the paediatric microbiome and the influence of early-life factors, including mode of delivery, breastfeeding, infections, antibiotic exposure, and environmental determinants, on immune programming. We then summarise evidence supporting the gut–lung axis in paediatric respiratory diseases and the gut–joint axis in juvenile idiopathic arthritis (JIA), highlighting recurrent but non-specific findings such as dysbiosis, impaired epithelial barrier integrity, altered microbial metabolite production, and immune dysregulation. Recent paediatric epidemiological evidence also showed that JIA was more prevalent among children with asthma than among those without asthma (0.81% vs. 0.23%), with asthma associated with approximately twofold higher odds of JIA after propensity-score weighting (OR 2.10, 95% CI 1.56–2.81), although this association does not establish microbiome-mediated causality. We finally critically examine whether more specific mechanisms, including gut-primed immune-cell trafficking, antigen-specific amplification, and metabolite–receptor convergence, could provide testable links between pulmonary and articular inflammation. On this basis, we propose the gut–lung–joint axis as a conceptual, hypothesis-generating model. Future research integrating microbiome profiling, immune-cell clonality, immunophenotyping, and metabolomics will be required to test its predictions and clarify its clinical relevance.

## 1. Introduction

The human body hosts a complex and dynamic community of microorganisms collectively known as the microbiome, which plays a fundamental role in maintaining physiological homeostasis and regulating immune function [1,2]. Among the various microbial ecosystems, the intestinal microbiota represents the largest and most metabolically active reservoir, exerting profound effects on host metabolism, epithelial barrier integrity, and immune maturation [2,3]. During childhood, the microbiome undergoes rapid development and remains particularly susceptible to environmental influences, making early life a critical period for immune programming and long-term disease susceptibility [4]. Over the past decade, increasing evidence has demonstrated that the effects of the gut microbiota extend far beyond the gastrointestinal tract. Through microbial metabolites, immune-cell trafficking, and systemic inflammatory mediators, intestinal microorganisms can influence distant organs and contribute to the regulation of immune responses throughout the body [1,2]. This concept has led to the emergence of the gut–lung axis, a bidirectional communication network linking intestinal and respiratory health. Alterations in gut microbial composition have been associated with impaired immune tolerance, chronic airway inflammation, and increased susceptibility to respiratory diseases, particularly asthma [4].

The clinical relevance of this field is underscored by the substantial burden of childhood asthma. A recent meta-analysis including 164 studies and 1,547,404 children estimated an overall childhood asthma prevalence of 10.2% (95% CI 9.5–11.0) [5].

Similarly, growing interest has focused on the role of the microbiome in rheumatic diseases. Paediatric rheumatic disorders are characterised by complex interactions among genetic susceptibility, environmental exposures, and immune dysregulation. In recent years, intestinal dysbiosis has been increasingly implicated in the pathogenesis of juvenile idiopathic arthritis (JIA) and other inflammatory joint disorders through mechanisms involving epithelial barrier dysfunction, altered microbial metabolites, and dysregulated T-cell responses, although no causal relation has been established [6,7].

JIA represents the most common chronic inflammatory rheumatic disease of childhood although epidemiological estimates vary substantially across populations and methodologies [8].

Although the gut–lung and gut–joint axes have traditionally been investigated as distinct biological entities, convergent microbiome-mediated mechanisms have been reported separately in respiratory and rheumatic diseases [6,7,9,10]. Shared mechanisms, including disruption of mucosal barriers, altered production of short-chain fatty acids (SCFAs), T helper 17/regulatory T-cell imbalance, trained immunity, and systemic dissemination of inflammatory mediators, suggest that the gut may influence immune processes relevant to both organ systems. However, the occurrence of similar microbiome-associated abnormalities in respiratory and rheumatic diseases does not itself demonstrate biological crosstalk between the lung and joint. The possibility of a broader gut–lung–joint axis has received limited attention, particularly in paediatric populations.

Although similar microbiome-mediated mechanisms have also been described in other inflammatory and immune-mediated diseases [4,11], respiratory and rheumatic disorders were selected because they represent two clinically distinct paediatric disease domains in which gut–organ crosstalk has been independently investigated. Their comparison is not intended to imply that general dysbiosis and associated mechanisms are specific to the lung or to the joint, but rather to determine whether evidence exists for more biologically specific processes capable of linking these compartments. In particular, gut-primed immune-cell trafficking, antigen-specific amplification, and metabolite–receptor convergence may provide candidate mechanisms through which independently described gut–lung and gut–joint relationships could be integrated into an experimentally testable framework.

Despite increasing evidence supporting the gut–lung and gut–joint axes independently, whether these relationships can be integrated through biologically specific mechanisms into a testable paediatric gut–lung–joint framework remains unresolved. To address this knowledge gap, this narrative review aims to (i) synthesise contemporary evidence on microbiome-mediated gut–lung and gut–joint interactions in childhood; (ii) distinguish shared, non-specific features of microbiome-associated dysregulation from mechanisms capable of supporting more specific interorgan crosstalk; and (iii) develop a conceptual, hypothesis-generating gut–lung–joint model based on clinical, epidemiological, experimental, and translational evidence. The proposed framework is intended to generate experimentally testable predictions.

## 2. Search Strategy

This study was conducted as a narrative review of evidence syntheses aimed at examining current knowledge on microbiome-mediated interactions between the gut, lungs, and joints in childhood. A literature search was performed in PubMed/MEDLINE for evidence syntheses published from 1 January 2020 to 18 June 2026. To broaden database coverage and update the evidence base, a supplementary targeted search was conducted in Scopus on 12 September 2026. Selected primary studies used separately for hypothesis development in Section 6 were not included in this count. No PRISMA workflow, protocol registration, or formal risk-of-bias or quality assessment was performed. Search concepts included combinations of keywords related to paediatric populations (“children”, “pediatric”, “paediatric”), the microbiome (“gut microbiota”, “gut microbiome”, “intestinal microbiota”, “respiratory microbiota”, “airway microbiota”, “lung microbiota”), microbiome-mediated interorgan communication (“gut–lung axis”, “gut–joint axis”), respiratory diseases (“asthma”, “wheezing”, “respiratory tract infection”, “bronchiolitis”), rheumatic diseases (“juvenile idiopathic arthritis”, “JIA”, “juvenile spondyloarthritis”, “spondyloarthritis”, “joint inflammation”), and evidence synthesis methodologies (“systematic review”, “narrative review”, “review” and “meta-analysis”). Search syntax was adapted to the requirements of each database. Only peer-reviewed articles published in English were considered.

The 2020 publication cutoff was applied to the evidence syntheses included in the formal literature selection to provide a contemporary overview of a rapidly evolving field and to focus the review on the current paediatric evidence base.

This restriction applied to the evidence syntheses included in the formal literature selection but not to seminal or mechanistically informative primary studies, which were eligible irrespective of publication year when required to establish biological plausibility or support the conceptual framework developed in Section 6.

Articles were eligible when they addressed at least one of the following topics: (i) gut–lung interactions or integrated gut and respiratory microbiota in children; (ii) microbiome alterations associated with paediatric respiratory or inflammatory joint diseases; (iii) early-life microbiome development and subsequent risk of respiratory or rheumatic disorders; (iv) microbiome-related mechanisms involved in immune regulation and joint inflammation, including JIA and juvenile spondyloarthritis (jSpA); or (v) microbiota-targeted interventions and their potential effects on respiratory or rheumatic diseases.

Articles were excluded when microbiota-related aspects represented only a marginal component of the article. Conference abstracts, study protocols, non-peer-reviewed publications, and articles not published in English were also excluded.

Selected primary clinical, epidemiological, experimental, and translational studies were considered separately from the formal evidence synthesis. Candidate studies were selected for Section 6 when they provided direct evidence relevant to: (i) gut-primed immune-cell generation, egress, or tissue recruitment; (ii) antigen-specific recognition or molecular mimicry; (iii) metabolite–receptor signalling relevant to pulmonary and articular inflammation; or (iv) intercompartmental T-cell clonality. These studies were used to establish biological plausibility and generate experimentally testable predictions rather than to provide a systematic or exhaustive synthesis of the primary literature.

Overall, 37 eligible evidence syntheses were included in the formal narrative synthesis, comprising 26 addressing the gut–lung/respiratory domain and 11 addressing the gut–joint/rheumatic domain. Their main characteristics are reported in Supplementary Tables S1 and S2. Selected primary studies used separately for hypothesis development in Section 6 were not included in this count.

## 3. Early-Life Determinants of Microbiome Development and Immune Programming

The establishment of the gut microbiome begins at birth and continues throughout the first years of life, representing a critical period for immune maturation and long-term health outcomes [4]. During this developmental window, microbial communities progressively evolve toward a more stable and diverse ecosystem dominated by the bacterial phyla Bacteroidetes and Firmicutes, alongside smaller proportions of Actinobacteria, Proteobacteria, and other taxa [2,4]. Beyond their metabolic functions, these microorganisms play an essential role in shaping mucosal and systemic immunity, promoting immune tolerance, maintaining epithelial barrier integrity, and regulating inflammatory responses [1,2].

Early microbial colonisation is influenced by multiple prenatal, perinatal, and environmental factors. Maternal conditions such as obesity, gestational diabetes, and dietary habits can alter maternal microbial communities and affect microbial transmission to the offspring [12,13,14]. Increasing evidence suggests that maternal microbial products and signals may influence fetal immune development before birth, supporting the concept that immune–microbial interactions may begin during prenatal life [15,16].

Mode of delivery represents one of the strongest determinants of neonatal microbiome composition. Vaginally delivered infants acquire microbial communities resembling maternal vaginal and intestinal microbiota, whereas cesarean-delivered infants are more frequently colonised by skin- and environment-associated microorganisms, resulting in delayed establishment of beneficial taxa such as *Bacteroides* and *Bifidobacterium* [3,17,18]. Similarly, prenatal, peripartum, and neonatal antibiotic exposure can disrupt microbial diversity and alter early immune programming, with potential long-term consequences for immune-mediated diseases, including asthma and allergic disorders [12,19,20].

Prematurity and prolonged neonatal hospitalisation further influence microbial development through reduced maternal microbial exposure, frequent antibiotic use, and intensive care-related environmental pressures [12,21]. These factors may contribute to persistent alterations in microbial composition and immune regulation during a particularly vulnerable stage of development.

Following birth, infant feeding and environmental exposures continue to shape microbiome maturation. Breastfeeding promotes the growth of beneficial microorganisms and provides bioactive compounds that support immune development [22]. The relevance of these early-life determinants is supported by longitudinal paediatric evidence. In a cohort of 440 children, 1453 faecal samples collected at 5, 13, 21, and 31 weeks and again at school age allowed characterisation of gut microbiota maturation from infancy to 6–11 years. Birth mode showed its strongest association with microbial composition during early infancy, with vaginal delivery associated with enrichment of *Bacteroides*, whereas from 13 weeks onward, diet—particularly breastfeeding cessation—became the principal determinant of microbial community structure. After adjustment for potential confounding factors, microbiota composition was also associated with the subsequent development of atopic dermatitis, allergic sensitization, and asthma, while members of the Lachnospiraceae family and the genera *Faecalibacterium* and *Dialister* were associated with a reduced risk of atopy. These findings might provide longitudinal support for a link between early-life exposures, microbiome maturation, and later immune-mediated outcomes [18]. Consistent with these observations, dietary patterns rich in fibre and plant-derived nutrients enhance microbial diversity and favour the production of SCFAs, including acetate, propionate, and butyrate, which exert anti-inflammatory effects, strengthen epithelial barrier function, and promote regulatory T-cell differentiation [2,23,24,25]. In contrast, microbial perturbations induced by antibiotics, dietary changes, urbanised lifestyles, or other environmental pressures may lead to dysbiosis, characterised by reduced microbial diversity and impaired host–microbiome homeostasis [1,2,4].

Collectively, these early-life determinants contribute to the establishment of microbiome configurations that may influence susceptibility to chronic inflammatory diseases later in life. The growing recognition that microbial signals can modulate immune responses beyond the intestinal tract might provide the biological foundation for the interconnected gut–lung and gut–joint pathways discussed in the following sections. The main early-life determinants and their potential consequences are summarised in Figure 1.

## 4. The Gut–Lung Axis in Paediatric Respiratory Diseases

### 4.1. Biological Basis of the Gut–Lung Axis

The gut–lung axis describes a bidirectional communication network through which intestinal microorganisms influence respiratory immunity and, conversely, pulmonary inflammation may affect intestinal homeostasis. This interaction is mediated by microbial metabolites, immune-cell trafficking, microbe-associated molecular patterns, and systemic inflammatory mediators that link mucosal sites across different organ systems [9,26].

Among these mechanisms, SCFAs, particularly acetate, propionate, and butyrate, represent the most extensively studied microbial mediators. Produced through bacterial fermentation of dietary fibres, SCFAs contribute to immune homeostasis by interacting with G-protein-coupled receptors, including GPR41/FFAR3 and GPR43/FFAR2, and by modulating gene expression through histone deacetylase inhibition [9]. Although present at relatively low concentrations within the respiratory tract, SCFAs produced in the gut can enter the systemic circulation and influence immune responses at distant sites, including the lungs. Experimental evidence suggests that propionate may regulate bone marrow haematopoiesis, dendritic-cell maturation, and allergic airway inflammation, supporting a mechanistic link between intestinal microbial activity and respiratory immune regulation [27].

Beyond microbial metabolites, the gut microbiota contributes to the development and maturation of both innate and adaptive immunity from the perinatal period onward. Increasing evidence indicates that early microbial colonisation shapes immune tolerance, influences T-cell differentiation, and contributes to the establishment of balanced inflammatory responses throughout life [10,28]. These mechanisms are increasingly recognised as central components of the gut–lung axis, particularly during early life, when microbiota-driven immune maturation may influence susceptibility to respiratory diseases [28,29,30,31].

Collectively, available evidence suggests that respiratory health is determined not only by the composition of the airway microbiota itself but also by the continuous interaction between intestinal microbial communities and systemic immune networks. Alterations in this finely regulated ecosystem may contribute to impaired immune tolerance, exaggerated inflammatory responses, and increased susceptibility to respiratory infections, recurrent wheezing, and asthma.

### 4.2. Early-Life Microbiome and Respiratory Disease Risk

Early life represents a critical window during which microbial colonisation, immune maturation, and exposure to respiratory pathogens occur simultaneously. Increasing evidence suggests that perturbations affecting the gut microbiota during this period may have long-term consequences for respiratory health and contribute to the development of recurrent wheezing and asthma [10,15,28,31,32].

Several longitudinal birth cohorts have demonstrated associations between early-life microbial composition and subsequent respiratory outcomes. Altered gut microbial profiles during infancy have been associated with an increased risk of recurrent wheezing and childhood asthma, particularly in genetically susceptible children. Differences in the abundance of genera such as *Bifidobacterium*, *Veillonella*, *Ruminococcus*, and members of the Lachnospiraceae family have been repeatedly reported in children who subsequently developed respiratory diseases [10]. Consistent with these findings, previous evidence syntheses have highlighted early-life dysbiosis, reduced microbial diversity, and impaired microbial maturation as potential contributors to asthma susceptibility [20,28,29,33].

The relationship between microbiota development and respiratory disease is likely influenced by multiple prenatal and postnatal factors, including maternal health, infectious exposures during pregnancy, mode of delivery, breastfeeding, antibiotic exposure, and environmental microbial contacts. Vaginal delivery and breastfeeding generally promote colonisation by beneficial microorganisms such as *Lactobacillus* and *Bifidobacterium*, whereas cesarean delivery and early antibiotic exposure have been associated with delayed microbial maturation and altered immune programming [2,10,28].

Maternal infections may represent an additional determinant of respiratory health trajectories in offspring. Evidence from a population-based prospective birth cohort demonstrated that maternal *Chlamydia trachomatis* infection during pregnancy was associated with an increased risk of wheezing and asthma, as well as impaired lung function in children up to 10 years of age [34]. Although the underlying mechanisms remain incompletely understood, maternal microbial and inflammatory exposures may influence fetal immune development and contribute to long-term susceptibility to respiratory diseases. Together with evidence linking maternal diet, antibiotic exposure, and mode of delivery to microbiome programming, these findings support the concept that respiratory disease risk may originate during early developmental stages, even before the establishment of a mature infant microbiome.

Respiratory viral infections represent an additional component of this complex interaction. Approximately one-third of children experiencing viral lower respiratory tract infections during infancy subsequently develop recurrent wheezing, and a proportion of these children later receive a diagnosis of asthma [29]. Respiratory syncytial virus (RSV) and rhinovirus infections have been most consistently associated with these outcomes, particularly among children with a personal or family history of atopy [35].

Emerging evidence suggests that the association between viral infections and later respiratory disease may be influenced in part by the microbiome. Infants who subsequently develop severe respiratory infections, recurrent wheezing, or asthma frequently exhibit distinct gut microbial profiles characterised by reduced abundance of potentially beneficial taxa, including *Bifidobacterium*, *Faecalibacterium*, *Roseburia*, and *Ruminococcus*, together with lower microbial diversity [27,29,33]. These findings support the hypothesis that microbial dysbiosis may influence antiviral immune responses and modify susceptibility to respiratory diseases.

The interaction between microbiota and respiratory infections appears to be bidirectional. On the one hand, pre-existing microbial communities may influence host immune responses and susceptibility to viral infections. On the other hand, respiratory viruses can induce changes in both airway and intestinal microbial ecosystems, potentially contributing to persistent immune dysregulation [27,35,36]. However, despite the consistency of these observations across multiple reports, current evidence remains largely observational, and direct causal relationships have yet to be conclusively demonstrated.

Collectively, available evidence indicates that respiratory disease risk is shaped by a complex interplay between early-life microbial colonisation, prenatal and postnatal environmental exposures, host immune development, and respiratory infections. These findings may support the concept of a gut–lung axis operating from the earliest stages of life and influencing susceptibility to respiratory diseases throughout childhood.

### 4.3. Microbiome Dysbiosis and Asthma Endotypes

Beyond influencing susceptibility to respiratory infections, the gut microbiota may also contribute to the development and maintenance of chronic airway inflammation. Increasing evidence suggests that asthma is associated with alterations in both intestinal and airway microbial communities, although the direction and causality of this relationship remain incompletely understood [10,37,38].

Several studies have reported reduced microbial diversity and compositional changes in children with asthma, including depletion of beneficial commensal bacteria and relative enrichment of potentially pro-inflammatory taxa. In particular, decreases in *Bacteroides* and SCFA-producing bacteria, together with increased abundance of *Proteobacteria*, have been associated with microbial profiles linked to airway inflammation and impaired immune regulation [37]. Similar observations have been reported in previous evidence syntheses examining the role of the gut microbiome in paediatric asthma [10,27,28,30,39].

Current concepts of asthma pathogenesis increasingly recognise the existence of distinct inflammatory endotypes. The most extensively studied is T2-high asthma, characterised by eosinophilic inflammation and increased expression of cytokines such as interleukin (IL)-4, IL-5, and IL-13.

T2-high asthma involves not only adaptive T helper 2 (Th2) responses but also innate type 2 immune pathways, particularly the activation of group 2 innate lymphoid cells (ILC2s), which contribute to the production of IL-5 and IL-13 [10]. Early-life microbial exposure may influence the maturation of the T helper 1 (Th1)/Th2 balance and, more broadly, type 2 immune programming [16]. Inadequate microbial stimulation during critical developmental windows may favour Th2-skewed adaptive responses, whereas appropriate microbial–immune interactions may support maturation toward a more balanced Th1/Th2 profile [30].

This endotypic distinction is also clinically relevant because current biologic therapies for severe paediatric asthma primarily target type 2 inflammatory pathways [40].

Recent research has reported associations between altered microbial profiles, reduced abundance of SCFA-producing bacteria, and asthma-related inflammatory phenotypes, although these findings remain predominantly observational [10,30]. Experimental evidence provides complementary mechanistic support for the ability of SCFAs to influence regulatory immune pathways and type 2 airway inflammation [10].

Through their effects on regulatory T cells, dendritic cells, and epithelial barrier function, microbial metabolites may contribute to maintaining immune tolerance and limiting allergic airway inflammation.

In contrast, non-T2 asthma may be characterised by neutrophilic inflammation and activation of innate immune pathways involving macrophages, neutrophils, and other innate immune cells. Although its relationship with the microbiome remains less clearly defined, emerging evidence suggests that dysbiosis may also influence these inflammatory pathways through altered microbial signalling, immune-cell activation, and persistent low-grade systemic inflammation [10,37,41].

Additional complexity arises from interactions between the microbiome and host genetic factors. Recent studies have suggested that host genetic variants may influence the relationship between microbial communities and asthma susceptibility [42]. For example, associations between the gut virome and childhood asthma appear to be modified by polymorphisms in genes involved in innate immune recognition, including Toll-like receptor 9 (*TLR9*) [42]. These observations support the concept that asthma results from dynamic interactions among microbial, immunological, and genetic factors rather than from isolated pathogenic mechanisms.

In summary, available evidence indicates that microbiome alterations may contribute not only to asthma susceptibility but also to disease heterogeneity. By influencing immune maturation, inflammatory pathways, and host–microbe interactions, the gut microbiota may participate in shaping different asthma endotypes and clinical phenotypes. However, most available evidence remains observational, and further longitudinal and mechanistic studies are needed to clarify causal relationships and identify microbiome-based biomarkers of disease risk and progression. The main microbiome-mediated mechanisms linking gut dysbiosis to paediatric respiratory outcomes are summarised in Figure 2.

## 5. The Gut–Joint Axis in Paediatric Rheumatic Diseases

### 5.1. Biological Basis of the Gut–Joint Axis

In recent years, increasing attention has focused on the potential role of intestinal microbiota in the pathogenesis of paediatric rheumatic diseases. Similar to the gut–lung axis, the gut–joint axis describes a bidirectional interaction between intestinal microbial communities and immune pathways involved in joint inflammation. Although the precise mechanisms remain incompletely understood, accumulating evidence suggests that alterations in gut microbial composition may contribute to the initiation and perpetuation of chronic inflammatory responses affecting the musculoskeletal system [6,43,44,45,46,47,48].

JIA, the most common chronic rheumatic disease in childhood, is currently regarded as a multifactorial disorder resulting from complex interactions among genetic susceptibility, environmental exposures, and immune dysregulation [43,44]. While infectious agents, dietary factors, and other environmental triggers have been investigated as potential contributors to disease onset, increasing evidence points to the intestinal microbiota as an important regulator of immune tolerance and inflammatory homeostasis [6,46,49].

The intestinal microbiota plays a central role in maintaining immune equilibrium through continuous interactions with epithelial cells, antigen-presenting cells, and lymphocyte populations [50]. Under physiological conditions, these interactions promote mucosal tolerance and limit excessive inflammatory responses. Conversely, disruption of microbial homeostasis may impair epithelial barrier integrity, alter microbial metabolite production, and facilitate systemic exposure to microbial products capable of activating both innate and adaptive immune pathways [6,51].

Several mechanisms have been proposed to explain how intestinal dysbiosis may influence joint inflammation. These include increased intestinal permeability, altered production of SCFAs, activation of innate immune receptors, molecular mimicry between microbial and host antigens, and dysregulation of T-cell responses, particularly involving the balance between regulatory T cells (Tregs) and pro-inflammatory T helper 17 (Th17) cells [7,48,51,52]. Notably, many of these pathways overlap with those previously described in the gut–lung axis, suggesting the existence of shared microbiome-mediated mechanisms across distinct organ systems.

Particular attention has been devoted to the IL-23/Th17 pathway, which is considered one of the most relevant immunological links between intestinal dysbiosis and chronic inflammatory diseases. Altered microbial communities may promote Th17-cell expansion and increased production of pro-inflammatory cytokines, including IL-17, IL-22, tumor necrosis factor (TNF)-α, and interferon (IFN)-γ, thereby contributing to synovial inflammation and tissue damage [7,48,51]. In parallel, reductions in SCFA-producing bacteria may impair regulatory immune pathways and further amplify inflammatory responses.

### 5.2. Microbiome Alterations in Juvenile Idiopathic Arthritis and Juvenile Spondyloarthritis

A growing body of evidence supports the existence of microbiome alterations in children with JIA and jSpA, although this second condition is still not clearly defined and not included in the International League of Associations for Rheumatology classification criteria [53,54].

Although a unique microbial signature has not yet been identified, multiple studies have consistently reported reduced microbial diversity, altered taxonomic composition, and depletion of bacteria involved in the maintenance of intestinal homeostasis [6,7,51,54,55,56,57].

Several reports have highlighted differences in gut microbial composition across JIA categories, suggesting that dysbiosis might contribute to disease heterogeneity [58]. In particular, reductions in SCFA-producing genera, including *Anaerostipes*, *Dialister*, *Lachnospira*, *Roseburia*, and *Faecalibacterium*, have been associated with increased disease activity and inflammatory burden [55]. In a matched cross-sectional study enrolling 40 children with JIA and 42 healthy controls, Qian et al. found significantly lower Chao1 and Shannon-Wiener diversity indices in JIA and reduced abundance of *Anaerostipes*, *Dialister*, *Lachnospira*, and *Roseburia*. A 12-genus random-forest classifier discriminated children with JIA from healthy controls with an area under the receiver operating characteristic curve of 0.798 [55]. These observations are of particular interest because SCFAs play a key role in maintaining epithelial barrier integrity and regulating anti-inflammatory immune responses.

Evidence also suggests that intestinal barrier dysfunction might represent a central component of the gut–joint axis. Increased intestinal permeability has been demonstrated in children with JIA and may facilitate the translocation of microbial products across the intestinal barrier [6,7]. This translocation might promote systemic immune activation and chronic inflammation through sustained exposure to microbial antigens and inflammatory mediators.

Further support for the gut–joint axis derives from the well-recognised relationship between inflammatory bowel disease (IBD) and rheumatic disorders. Children with JIA exhibit a higher prevalence of IBD than the general paediatric population, while arthritis represents the most common extraintestinal manifestation of IBD [6]. These observations suggest shared pathogenic mechanisms linking intestinal and joint inflammation.

The association appears particularly strong in jSpA and enthesitis-related arthritis (ERA), conditions in which subclinical intestinal inflammation is frequently observed [59]. Up to 50% of patients with jSpA might exhibit microscopic intestinal inflammation, while a substantial proportion of patients with IBD develop musculoskeletal manifestations [6,54]. In both adult and paediatric populations, alterations involving *Dialister*, *Ruminococcus gnavus*, *Bacteroides fragilis*, and reductions in beneficial SCFA-producing bacteria have been reported, although findings remain heterogeneous across studies [54].

Clinical studies have reported associations between dysbiosis and inflammatory disease features in JIA and jSpA, but they do not establish whether microbiome alterations precede disease onset or arise as a consequence of ongoing inflammation [6,7,51,54]. Experimental studies provide complementary mechanistic evidence that altered microbial metabolism, epithelial barrier dysfunction, and pro-inflammatory immune activation may contribute to joint inflammation [7,51,52].

Nevertheless, considerable interindividual variability persists, and no single microbial profile has yet demonstrated sufficient consistency to serve as a diagnostic biomarker.

Overall, current evidence might support the presence of significant microbiome alterations in paediatric inflammatory joint diseases and the biological plausibility of a gut–joint axis. However, the heterogeneity of available studies highlights the need for larger longitudinal investigations capable of clarifying causal relationships and identifying reproducible microbiome-associated disease signatures.

### 5.3. Immune Mechanisms Linking Gut Dysbiosis and Joint Inflammation

Although the precise relationship between intestinal dysbiosis and paediatric rheumatic diseases remains incompletely understood, accumulating evidence might suggest that alterations in microbial composition might contribute to the initiation and perpetuation of chronic joint inflammation through multiple interconnected immune pathways [7,51,52].

One of the most widely investigated mechanisms involves disruption of intestinal barrier integrity. Under physiological conditions, the intestinal epithelium acts as a selective barrier that regulates interactions between luminal microorganisms and the host immune system. Dysbiosis may impair this barrier through alterations in mucosal homeostasis, increased zonulin production, and disruption of tight junction proteins, resulting in increased intestinal permeability, commonly referred to as a “leaky gut” [6,7,51]. This process might facilitate the translocation of microbial products, including lipopolysaccharides and other pathogen-associated molecular patterns, into the systemic circulation, thereby promoting immune activation beyond the intestinal compartment.

Microbial translocation can stimulate innate immune receptors such as Toll-like receptors (TLRs) and NOD-like receptors (NLRs), leading to the production of pro-inflammatory cytokines including TNF-α, IL-1β, IL-6, and IFN-γ [7,51]. Sustained activation of these pathways may contribute to the chronic inflammatory environment characteristic of juvenile inflammatory arthritis. In parallel, microbial antigens may induce molecular mimicry, whereby immune responses directed against bacterial components cross-react with host tissues, potentially contributing to the loss of self-tolerance [52].

A central role has been attributed to the imbalance between regulatory and pro-inflammatory T-cell populations [50]. Several studies suggest that dysbiosis may favour Th17-cell expansion and activation of the IL-23/IL-17 inflammatory axis, while simultaneously impairing Treg function [7,51,52]. Increased production of IL-17, IL-22, and TNF-α promotes recruitment and activation of inflammatory cells within the synovium, contributing to persistent joint inflammation and tissue damage. Conversely, Tregs play a fundamental role in maintaining immune tolerance and limiting excessive inflammatory responses.

Microbial metabolites represent another critical link between intestinal microorganisms and systemic immunity. SCFAs, particularly butyrate, acetate, and propionate, exert anti-inflammatory effects through several mechanisms, including enhancement of epithelial barrier integrity, modulation of dendritic-cell function, and promotion of Treg differentiation through G-protein-coupled receptor signalling and histone deacetylase inhibition [7,52,57]. Reduced abundance of SCFA-producing bacteria, frequently reported in JIA and jSpA, might therefore contribute to impaired immune regulation and increased susceptibility to chronic inflammation.

Specific microbial species may further influence immune homeostasis. For example, *Bacteroides fragilis* produces polysaccharide A, a molecule capable of promoting IL-10-producing FOXP3+ regulatory T-cell responses and supporting immune tolerance. In contrast, enrichment of potentially pro-inflammatory taxa and pathobionts may enhance innate immune activation and perpetuate systemic inflammation [7,54].

Importantly, these immunological alterations are not confined to the intestinal environment. Activated immune cells, circulating cytokines, and microbially derived metabolites may reach distant tissues, including the synovium, where they may contribute to inflammatory processes. This systemic dissemination of microbiome-mediated immune signals might provide a plausible biological explanation for the association between intestinal dysbiosis and paediatric rheumatic diseases.

Collectively, current evidence might be consistent with a model in which intestinal dysbiosis, epithelial barrier dysfunction, altered microbial metabolite production, and immune-cell dysregulation interact to promote chronic joint inflammation. Notably, many of these mechanisms, including epithelial barrier disruption, Th17/Treg imbalance, microbial metabolite signalling, and systemic immune activation, closely resemble those described in the gut–lung axis, suggesting the existence of shared microbiome-mediated pathways linking respiratory and rheumatic diseases. These mechanisms are summarised in Figure 3.

## 6. From Shared Dysbiosis to an Experimentally Testable Gut–Lung–Joint Axis

The gut–lung and gut–joint axes share several microbiome-associated alterations, summarised in Table 1. However, these convergent features are not organ-specific and cannot, by themselves, establish a distinct gut–lung–joint axis. Throughout this section, evidence is distinguished according to its source as paediatric human, adult human, experimental animal, or hypothetical cross-context integration, as indicated in Table 2 and Figure 4.

The convergence of microbiome-associated alterations in respiratory and rheumatic diseases does not, by itself, establish a distinct gut–lung–joint axis. Reduced microbial diversity, impaired epithelial barrier integrity, altered microbial metabolite production, and Th17/Treg imbalance are common features of several immune-mediated and inflammatory disorders and therefore lack organ specificity. The biological relevance of the proposed axis must therefore rely on mechanisms capable of linking defined intestinal microbial signals with pulmonary and articular inflammation in a more specific and experimentally testable manner.

Importantly, recent paediatric epidemiological evidence might provide direct clinical support for an association between respiratory and rheumatic diseases. Lee et al. analysed data from 169,786 children and adolescents aged 0–17 years participating in six cycles of the U.S. National Survey of Children’s Health (2016–2021). JIA was more prevalent among children with asthma than among those without asthma (0.81% vs. 0.23%). After propensity-score weighting for demographic, perinatal, socioeconomic, and environmental covariates, asthma remained associated with approximately twofold higher odds of JIA (OR 2.10, 95% CI 1.56–2.81), with a similar estimate obtained after additional adjustment for age, sex, and race/ethnicity (OR 2.06, 95% CI 1.53–2.76). These findings might provide population-level evidence of a paediatric lung–joint association, although the cross-sectional design precludes inference regarding temporality or causality and the study did not investigate microbiome-mediated mechanisms [60].

Experimental autoimmune arthritis models may provide proof-of-principle that intestinal microbial signals can generate immune-cell populations with systemic consequences. In the K/BxN autoimmune arthritis model, segmented filamentous bacteria (SFB) promote intestinal Th17 responses and accelerate autoimmune arthritis. Subsequent studies showed that SFB-driven immune programming is not restricted to the intestinal compartment: T follicular helper (Tfh) and Tfh17 populations arising within Peyer’s patches can reach systemic lymphoid sites, where they promote germinal-center responses and autoantibody production. More recently, sphingosine-1-phosphate receptor 1 (S1PR1) was identified as one mechanism facilitating the egress of gut-derived Tfh17 cells, providing a mechanistic basis for the systemic dissemination of microbiota-primed lymphocytes [61,62,63].

Tissue recruitment may represent a second step in this process. Bradley et al. demonstrated that SFB-induced intestinal Th17 cells were preferentially recruited to the lung in the K/BxN model, where the inflammatory environment was characterised by high expression of C-C motif chemokine ligand 20 (CCL20) [64]. Because Th17 cells express their receptor C-C chemokine receptor type 6 (CCR6), these findings support a model in which gut-derived immune-cell expansion can be followed by selective recruitment into an inflamed pulmonary environment. A potentially convergent mechanism has been identified in paediatric arthritis. In children with JIA, Nistala et al. showed that IL-17-producing CD4+ cells were enriched in inflamed joints, expressed CCR6, and migrated preferentially toward CCL20 [65]. Thus, although CCR6–CCL20 is neither lung- nor joint-specific and identical gut-derived Th17 clones have not been demonstrated in both tissues, this pathway represents a plausible shared recruitment mechanism. Within the proposed model, S1PR1-dependent egress from gut-associated lymphoid tissues followed by CCR6–CCL20–associated tissue recruitment may therefore constitute sequential, experimentally testable steps linking intestinal immune priming with distant inflammatory sites.

Antigen-specific recognition may provide an additional level of biological selectivity. In the same experimental model, Bradley et al. showed that SFB preferentially expanded dual-T-cell receptor (TCR) Th17 cells capable of combining recognition of a commensal antigen with self-reactivity [64]. This mechanism differs from classical molecular mimicry and may provide proof-of-principle that exposure to a defined intestinal microbial antigen can selectively amplify an autoreactive T-cell population rather than inducing only nonspecific systemic inflammation.

A complementary antigen-specific mechanism has been described in human rheumatoid arthritis (RA). Pianta et al. identified sequence homology between microbial peptides derived from intestinal commensals and the synovial autoantigens N-acetylglucosamine-6-sulfatase (GNS) and filamin A (FLNA). T-cell responses to these self-peptides were associated with responses to the corresponding microbial peptides, supporting molecular mimicry as a potential mechanism linking intestinal microbial immunity with joint autoimmunity [66]. However, these observations derive from adults with RA, and no gut microbial antigen has yet been shown to cross-react with autoantigens shared specifically by the lung and joint. Antigen-specific amplification should therefore be regarded as a biologically plausible component of the proposed axis rather than evidence of an established cross-reactive mechanism specific to the lung and joint.

Metabolite–receptor signalling offers a third possible route of convergence. Succinate is particularly informative because the same metabolite-receptor system has independently been implicated in pulmonary and articular inflammation. Wang et al. demonstrated that gut microbiota-derived succinate can accumulate in the lung and promote inflammatory responses through succinate receptor 1 (SUCNR1) on alveolar macrophages [67]. Independently, Littlewood-Evans et al. showed that extracellular succinate–SUCNR1/GPR91 signalling amplifies macrophage activation and IL-1β production in experimental arthritis, while succinate is abundant in synovial fluid from patients with RA [68]. These findings do not indicate that gut-derived succinate selectively targets the lung and joint, nor has intestinally derived succinate been shown to simultaneously drive inflammation in both compartments. Rather, they demonstrate that the same metabolite–receptor pair can become functionally relevant within distinct inflammatory microenvironments. Tissue convergence may therefore depend on context-dependent metabolite availability, receptor expression, and activation of resident immune cells rather than on exclusive organ-specific receptor expression.

Taken together, these observations suggest that the proposed gut–lung–joint axis may involve three complementary levels of biological specificity: immune-cell trafficking, through the generation, egress, and tissue recruitment of gut-primed Th17/Tfh17-lineage cells; antigen-specific amplification, through dual-TCR recognition or molecular mimicry; and metabolite–receptor convergence, exemplified by succinate–SUCNR1 signalling. Importantly, none of these mechanisms currently demonstrates the existence of a unified gut–lung–joint pathway in humans, and much of the mechanistic evidence derives from experimental arthritis models or adult inflammatory disease. Their value therefore lies primarily in providing biologically grounded mechanisms from which testable predictions can be generated.

A critical prediction of the proposed axis is that clonally related immune-cell populations should be identifiable across anatomically distinct compartments within the same individual. The feasibility of this approach is supported by Venken et al., who identified identical TCR clonotypes in paired lung and synovial samples from adults with early anti-citrullinated protein antibody (ACPA)-positive RA, with many of these clonotypes also detectable in the circulation [69]. Although this study investigated a smoking-associated lung–joint interaction rather than intestinal immunity, it demonstrates that pulmonary and articular inflammatory compartments can contain clonally related T-cell populations. In paediatric disease, Rossetti et al. identified shared Treg clonotypes between peripheral blood and synovial fluid in JIA, supporting the feasibility of TCR-based tracking of tissue-associated lymphocyte populations in children [70].

Future studies should therefore test the proposed axis through longitudinal, multi-compartment approaches integrating microbiome profiling, TCR sequencing, immunophenotyping, chemokine-receptor analysis, antigen-specific functional assays, and metabolomics. Evidence supporting the model would include temporal precedence of gut microbial and immune alterations, reproducible clonal relationships among intestinal, circulating, pulmonary, and synovial T-cell populations, compatible trafficking phenotypes, and coordinated responses to manipulation of candidate pathways. Conversely, the absence of reproducible intercompartmental clonal relationships, failure to demonstrate temporal precedence of gut-associated immune alterations, or failure of targeted disruption of the proposed trafficking, antigen-recognition, or metabolite–receptor pathways to modify both pulmonary and articular inflammation would argue against a functionally integrated gut–lung–joint axis.

The principal candidate mechanisms, their supporting evidence, and the corresponding testable and falsifiable predictions are summarised in Table 2.

**Table 2:** Testable hypotheses derived from the proposed gut–lung–joint axis.

Candidate Mechanism	Supporting Evidence/Source	Test/Evidence Against
Gut-primed immune-cell trafficking [61,62,63,64,65]	Th17/Tfh17 egress; CCR6–CCL20 recruitment (experimental animal/paediatric human)	TCR tracking across compartments; no reproducible clonal/trafficking pattern argues against
Antigen-specific amplification [64,66]	Dual-TCR recognition; microbial–self cross-reactivity (experimental animal/adult human)	Antigen-specific T-cell assays; no reproducible cross-reactivity argues against
Metabolite–receptor convergence [67,68]	Succinate–SUCNR1 in lung and arthritis (experimental animal/adult human)	Metabolomics/receptor blockade; no coordinated pathway effect argues against
Intercompartmental T-cell clonality [69,70]	Lung–synovium RA; blood–synovium JIA clonotypes (adult/paediatric human)	Multi-compartment TCR sequencing; no reproducible clonal overlap argues against

Abbreviations: CCL20, C-C motif chemokine ligand 20; CCR6, C-C chemokine receptor type 6; JIA, juvenile idiopathic arthritis; RA, rheumatoid arthritis; SUCNR1, succinate receptor 1; TCR, T-cell receptor; Tfh17, T follicular helper 17; Th17, T helper 17.

The proposed gut–lung–joint axis should therefore be regarded as a conceptual, hypothesis-generating and experimentally testable model, rather than as an established biological pathway (Figure 4).

## 7. Limitations of the Present Review and Available Evidence

Several limitations should be considered when interpreting the findings of this review. The literature search was restricted to English-language publications and was not conducted according to a formal systematic-review protocol. No formal risk-of-bias or quality assessment was performed. The non-systematic selection of primary mechanistic studies may also have introduced selection or confirmation bias, because these studies were selected for their relevance to the candidate mechanisms rather than through a systematic search of the primary literature.

Because multiple included articles may synthesise overlapping primary studies, recurrence of a finding across articles should not be interpreted as independent replication of the underlying evidence.

The evidence supporting the proposed gut–lung–joint axis remains largely indirect. Although substantial evidence supports the existence of both the gut–lung and gut–joint axes, studies simultaneously investigating intestinal, respiratory, and musculoskeletal outcomes in paediatric populations are currently lacking. Candidate linking mechanisms, including gut-primed Th17/Tfh17-cell trafficking, CCR6–CCL20–associated tissue recruitment, antigen-specific amplification, and succinate–SUCNR1 signalling, derive from different experimental models, adult disease contexts, or separate paediatric observations. No study has yet demonstrated these candidate mechanisms as a coordinated gut–lung–joint pathway. Although recent paediatric epidemiological evidence supports an association between asthma and JIA, no paediatric study has yet demonstrated a microbiome-mediated, integrated gut–lung–joint pathway.

The available literature is characterised by considerable heterogeneity in study design, microbiome assessment methods, patient populations, disease definitions, and analytical approaches. This variability limits direct comparisons across studies and may contribute to inconsistencies in the identification of disease-specific microbial signatures.

## 8. Future Directions and Research Priorities

Despite the growing interest in microbiome-mediated interactions in childhood diseases, several important questions remain unresolved. A major challenge in both the gut–lung and gut–joint fields is determining whether dysbiosis acts as a primary driver of disease development, a consequence of ongoing inflammation, or a combination of both. Most available studies are observational and frequently involve heterogeneous populations, different sampling strategies, and variable microbiome analysis methods, making comparisons across studies difficult.

Another important challenge is the lack of standardised microbial signatures consistently associated with paediatric respiratory or rheumatic diseases. Future research should prioritise large longitudinal paediatric cohorts beginning during pregnancy or early infancy [49]. Such studies might help clarify the temporal relationship between microbiome development, immune maturation, and the subsequent onset of respiratory and rheumatic diseases. Simultaneous assessment of environmental exposures, microbial composition, immune responses, and clinical outcomes will be essential to identify critical windows of susceptibility and potential causal pathways.

Particular attention should be devoted to studies capable of investigating respiratory, intestinal, and musculoskeletal outcomes within the same population. To date, gut–lung and gut–joint interactions have largely been explored independently. Evaluating these pathways within integrated research frameworks may help determine whether they represent components of a biologically integrated gut–lung–joint axis rather than distinct biological entities or organ-specific associations.

At present, the proposed gut–lung–joint framework should be regarded primarily as a research framework for generating and testing mechanistic hypotheses rather than as a clinically validated tool for diagnosis, risk stratification, biomarker selection, or therapeutic decision-making.

Future clinical trials should be able to evaluate, along with clinical outcomes, long-term microbiome changes. Factors such as age, baseline microbial composition, environmental exposures, treatment duration, and safety profiles should be carefully considered. While microbiota-targeted interventions, including dietary approaches, probiotics, prebiotics, and faecal microbiota transplantation, remain promising, current evidence is insufficient to support their routine implementation in paediatric respiratory or rheumatic diseases.

## 9. Conclusions

Growing evidence indicates that the gut microbiome may play a role in shaping immune development and maintaining immune homeostasis throughout childhood. Research conducted over the last decade has increasingly highlighted communication between the gut and the lungs, as well as associations between the gut and the joints, mediated by microbial, metabolic, and immunological pathways.

Although the evidence supporting the gut–lung axis is currently more robust than that available for the gut–joint axis, both fields share several common biological mechanisms, including alterations in microbial diversity, impaired epithelial barrier function, dysregulated production of microbial metabolites, and imbalance between pro-inflammatory and regulatory immune responses. These observations suggest that respiratory and rheumatic diseases, while remaining clinically distinct entities, may share components of broader microbiome-mediated immune dysregulation. Therefore, we propose the gut–lung–joint axis as a conceptual, hypothesis-generating model whose rationale extends beyond shared mechanisms to include candidate processes with greater biological specificity, including gut-primed Th17/Tfh17-cell trafficking, antigen-specific amplification, and metabolite–receptor convergence. However, these mechanisms have not been demonstrated as a unified pathway in children, and validation will require longitudinal, multi-compartment paediatric studies.

A deeper understanding of these potentially interconnected pathways may ultimately inform the future development and evaluation of personalised approaches to the prevention and management of paediatric respiratory and rheumatic diseases.

## Figures and Tables

**Figure 1:**
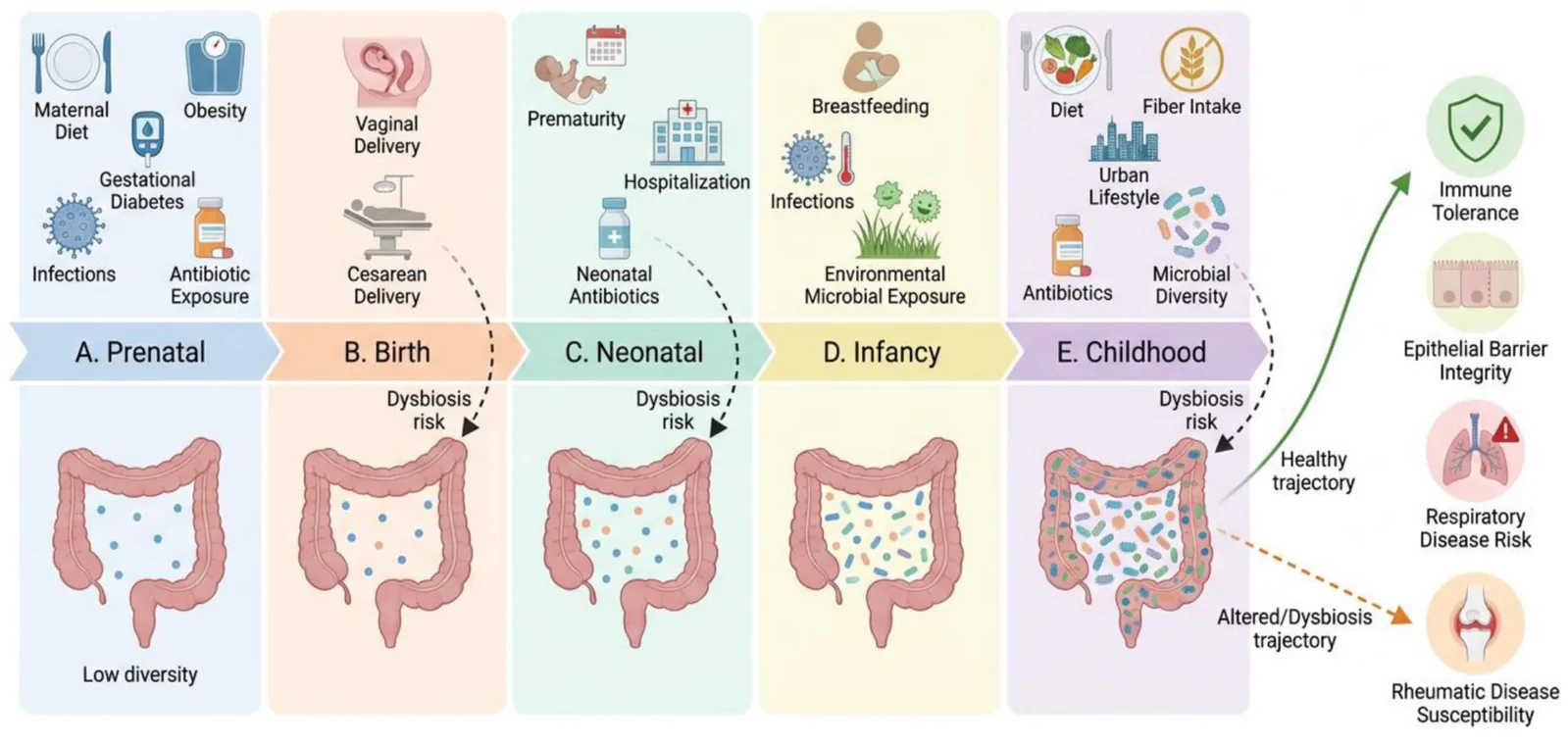
**Early-life determinants of gut microbiome development and immune programming in childhood.** Prenatal, perinatal, and postnatal exposures shape microbial maturation. Balanced microbiome development supports epithelial barrier integrity and immune tolerance, whereas microbiome disruption may promote dysbiosis and contribute to susceptibility to paediatric respiratory and rheumatic diseases. Created by the authors using FigureLabs, version: Starter-251225, 2026, Basel, Switzerland.

**Figure 2:**
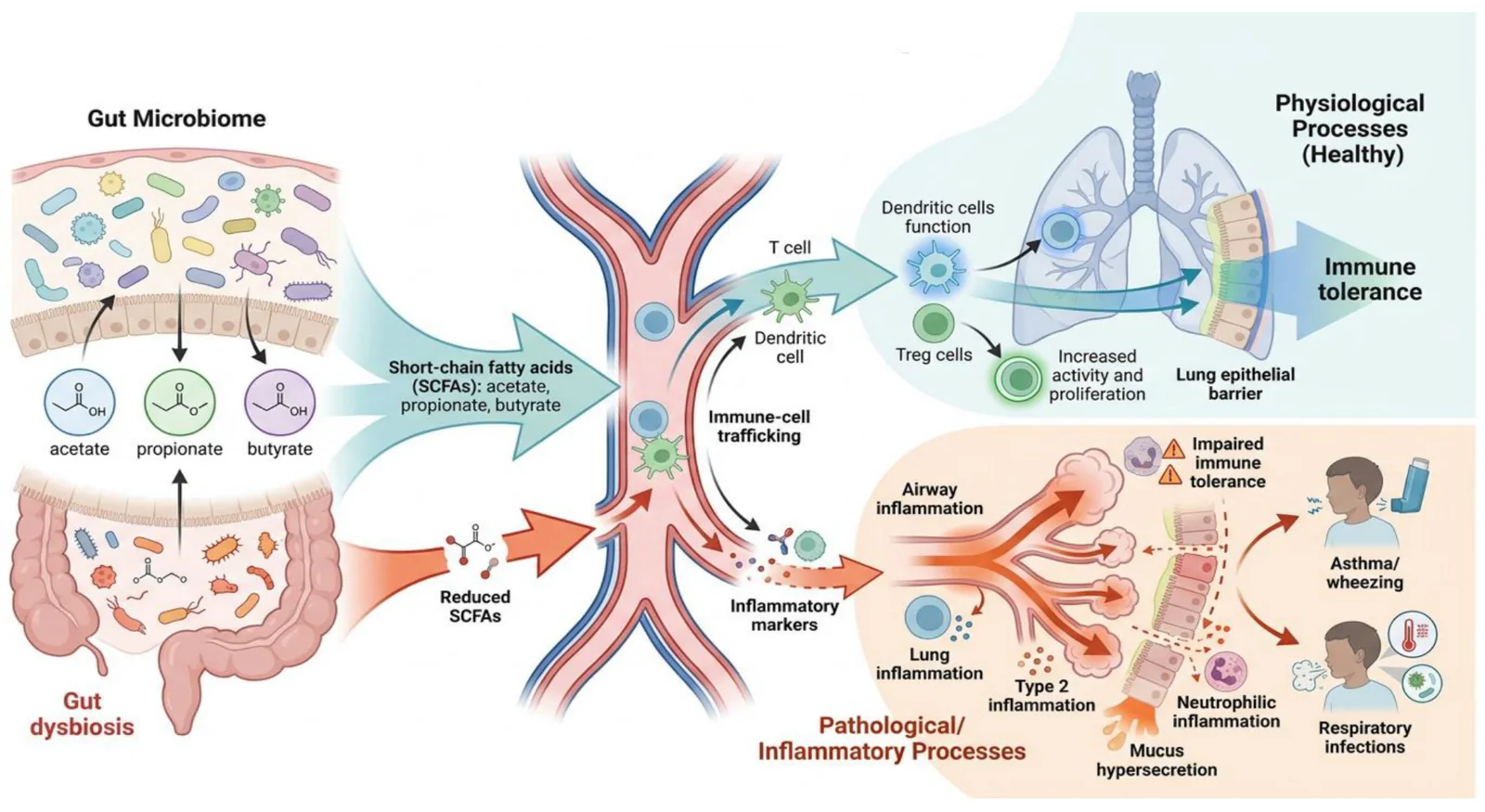
**Paediatric gut–lung axis in health and disease**. Gut-derived microbial metabolites contribute to respiratory immune homeostasis by supporting epithelial barrier integrity and immune tolerance. In contrast, gut dysbiosis and reduced SCFA availability may promote systemic and airway inflammation, increasing susceptibility to asthma, wheezing, and respiratory infections. Created by the authors using FigureLabs, version: Starter-251225, 2026, Basel, Switzerland. Abbreviation: SCFA, short-chain fatty acid.

**Figure 3:**
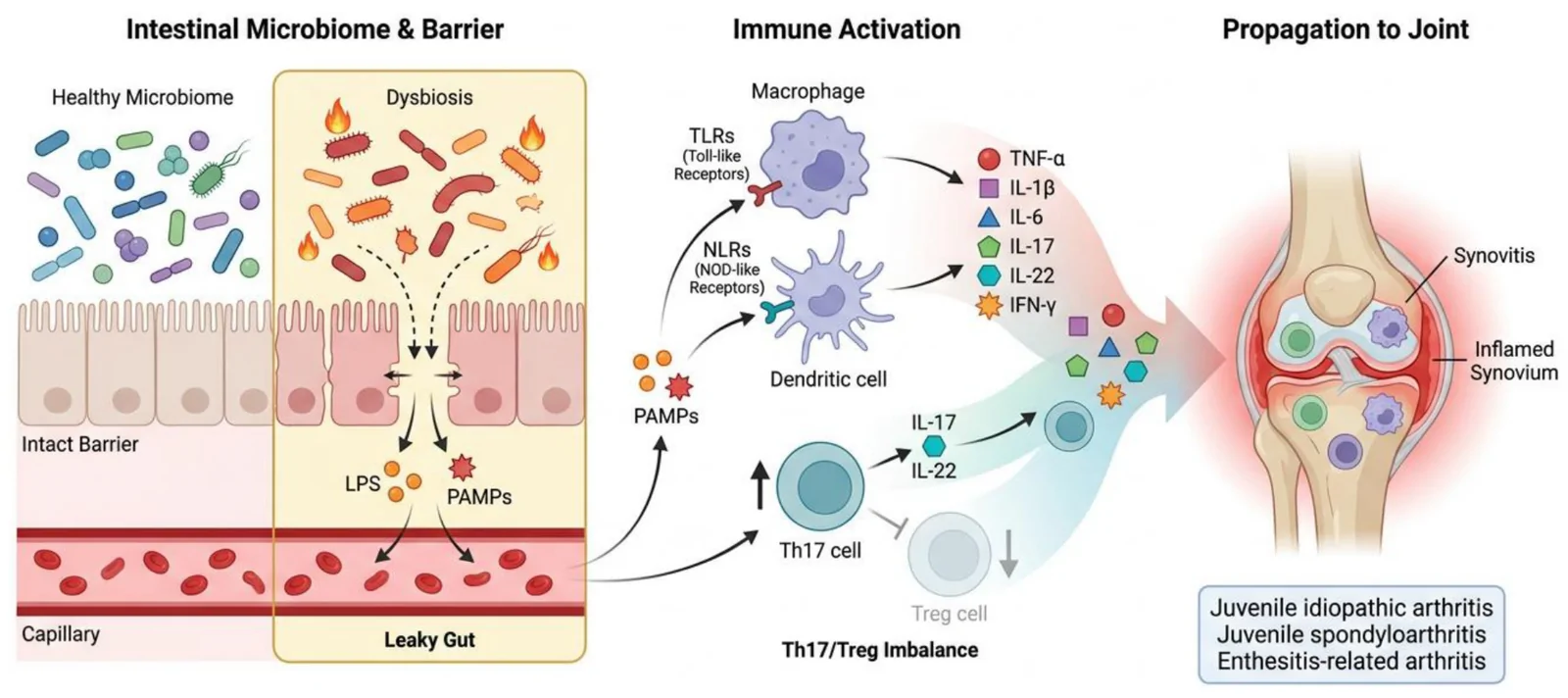
**Gut–joint axis in paediatric rheumatic diseases.** Gut dysbiosis and impaired intestinal barrier integrity may facilitate the systemic translocation of microbial products, activating innate immune pathways and pro-inflammatory cytokine responses. The resulting Th17/Treg imbalance and systemic immune activation may contribute to synovial and enthesis inflammation in juvenile idiopathic arthritis, juvenile spondyloarthritis, and enthesitis-related arthritis. Created by the authors using FigureLabs, version: Starter-251225, 2026, Basel, Switzerland. Abbreviations: IFN, interferon; IL, interleukin; LPS, lipopolysaccharide; NLR, NOD-like receptor; PAMP, pathogen-associated molecular pattern; Th17, T helper 17; TLR, Toll-like receptor; TNF, tumor necrosis factor; Treg, regulatory T cell.

**Figure 4:**
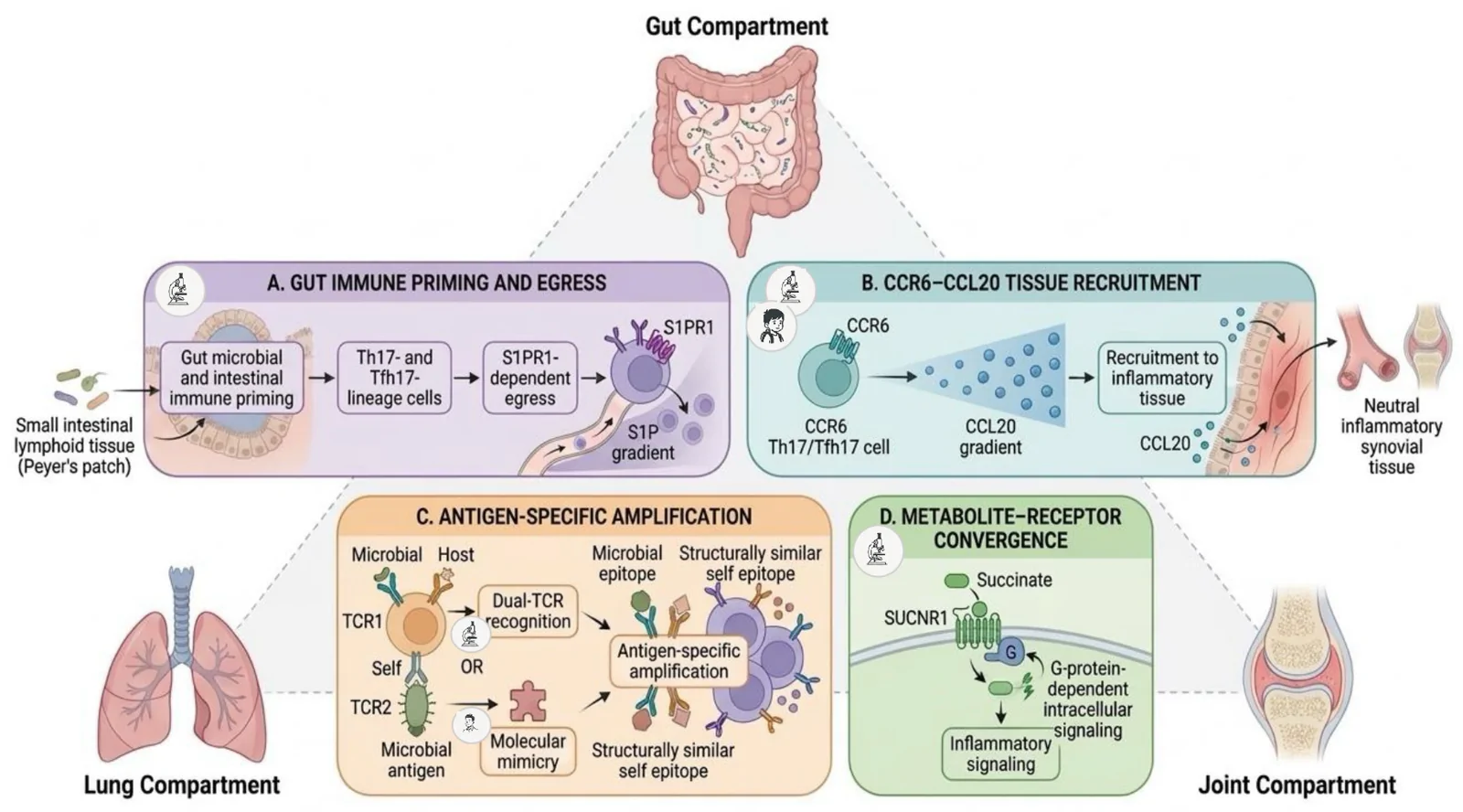
Conceptual, hypothesis-generating model of the proposed paediatric gut–lung–joint axis. Candidate mechanisms extending beyond general dysbiosis include gut priming of Th17/Tfh17-lineage cells and S1PR1-dependent egress, CCR6–CCL20–associated recruitment to pulmonary and articular inflammatory environments, antigen-specific amplification through dual-TCR recognition or molecular mimicry, and metabolite–receptor convergence exemplified by succinate–SUCNR1 signalling. These mechanisms are supported in distinct experimental or clinical contexts but have not been demonstrated as a unified tri-compartmental pathway. Solid arrows denote individual relationships supported by experimental or clinical evidence, whereas dashed connectors indicate hypothetical integration across compartments. Icons indicate the source of supporting evidence: the microscope denotes experimental animal evidence, the child silhouette denotes paediatric human evidence, and the adult silhouette denotes adult human evidence. Created by the authors using FigureLabs, version: Starter-251225, 2026, Basel, Switzerland. Abbreviations: CCL20, C-C motif chemokine ligand 20; CCR6, C-C chemokine receptor type 6; S1PR1, sphingosine-1-phosphate receptor 1; SUCNR1, succinate receptor 1; TCR, T-cell receptor; Tfh17, T follicular helper 17; Th17, T helper 17.

**Table 1:** Shared but non-specific gut microbiome features reported in paediatric respiratory and rheumatic diseases.

Shared Microbiome Feature	Suggested Evidence in Paediatric Respiratory Diseases	Suggested Evidence in Paediatric Rheumatic Diseases	Potential Shared Immunological Pathways
Reduced gut microbial diversity	Associated with asthma, wheezing, RTIs [20,28,29,33,37]	Associated with JIA [6,7,54,56] and jSpA	Reduced microbial resilience may impair immune homeostasis
Reduced SCFA-producing bacteria *	Associated with increased asthma risk and airway inflammation [10,27,28,30,37]	Associated with increased inflammatory activity [6,7,51,54,55]	Reduced SCFAs may contribute to barrier dysfunction and reduced Treg differentiation
Altered *Bacteroides* spp.	Early-life dysbiosis and asthma [10,27,30,37]	Associated with JIA and jSpA [6,7,54,56]	Altered immune tolerance
Increased pro-inflammatory taxa †	Increased airway inflammation [10,27,37,38]	Associated with jSpA and IBD-associated arthritis [6,7,51,54]	Increased innate activation may contribute to systemic inflammation

Abbreviations: SCFAs, short-chain fatty acids; RTIs, respiratory tract infections; JIA, juvenile idiopathic arthritis; jSpA, juvenile spondyloarthritis; IBD, inflammatory bowel disease; Treg, regulatory T cells. * Including *Faecalibacterium*, *Roseburia*, *Lachnospira*, *Anaerostipes*, and *Dialister*. † Examples include Enterobacteriaceae and *Ruminococcus gnavus*; the specific taxa reported vary according to disease and study population.

## Data Availability

No new data were created or analysed in this study. Data sharing is not applicable to this article.

## Supplementary Materials

The following supporting information can be downloaded at: https://www.mdpi.com/article/10.3390/ijms27198692/s1, References [71,72,73,74] are cited in Supplementary Table S1.

## References

[1] Zheng D., Liwinski T., Elinav E. (2020). Interaction between Microbiota and Immunity in Health and Disease. Cell Res..

[2] Fusco W., Lorenzo M.B., Cintoni M., Porcari S., Rinninella E., Kaitsas F., Lener E., Mele M.C., Gasbarrini A., Collado M.C. (2023). Short-Chain Fatty-Acid-Producing Bacteria: Key Components of the Human Gut Microbiota. Nutrients.

[3] Suárez-Martínez C., Santaella-Pascual M., Yagüe-Guirao G., Martínez-Graciá C. (2023). Infant Gut Microbiota Colonization: Influence of Prenatal and Postnatal Factors, Focusing on Diet. Front. Microbiol..

[4] Bankole T., Li Y. (2025). The Early-Life Gut Microbiome in Common Pediatric Diseases: Roles and Therapeutic Implications. Front. Nutr..

[5] Zhou W., Tang J. (2025). Prevalence and Risk Factors for Childhood Asthma: A Systematic Review and Meta-Analysis. BMC Pediatr..

[6] Matei D.E., Rosser E.C. (2026). The Gut-Joint Axis in Juvenile Idiopathic Arthritis. Arthritis Res. Ther..

[7] Xin L., He F., Li S., Zhou Z.-X., Ma X.-L. (2021). Intestinal Microbiota and Juvenile Idiopathic Arthritis: Current Understanding and Future Prospective. World J. Pediatr..

[8] Costa Dias S., Habre C., Di Paolo P.L., d’Angelo P., Augdal T.A., Angenete O.W., Kljucevsek D., Inarejos Clemente E.J., Tanturri De Horatio L., Rosendahl K. (2025). ESR Essentials: Juvenile Idiopathic Arthritis; What Every Radiologist Needs to Know—Practice Recommendations by the European Society of Paediatric Radiology. Eur. Radiol..

[9] Stricker S., Hain T., Chao C.-M., Rudloff S. (2022). Respiratory and Intestinal Microbiota in Pediatric Lung Diseases—Current Evidence of the Gut–Lung Axis. Int. J. Mol. Sci..

[10] Liu L., Zhao W., Zhang H., Shang Y., Huang W., Cheng Q. (2025). Relationship between Pediatric Asthma and Respiratory Microbiota, Intestinal Microbiota: A Narrative Review. Front. Microbiol..

[11] Sadeghpour Heravi F. (2024). Gut Microbiota and Autoimmune Diseases: Mechanisms, Treatment, Challenges, and Future Recommendations. Curr. Clin. Microbiol. Rep..

[12] Coscia A., Bardanzellu F., Caboni E., Fanos V., Peroni D.G. (2021). When a Neonate Is Born, So Is a Microbiota. Life.

[13] Ionescu R.F., Enache R.M., Cretoiu S.M., Gaspar B.S. (2022). Gut Microbiome Changes in Gestational Diabetes. Int. J. Mol. Sci..

[14] Soderborg T.K., Carpenter C.M., Janssen R.C., Weir T.L., Robertson C.E., Ir D., Young B.E., Krebs N.F., Hernandez T.L., Barbour L.A. (2020). Gestational Diabetes Is Uniquely Associated with Altered Early Seeding of the Infant Gut Microbiota. Front. Endocrinol..

[15] Wang Y., Yu J., Wang Y., Li Y. (2026). Early-Life Microbiota Exposure and Prevention of Childhood Asthma: Immunological Mechanisms and Intervention Perspectives. Front. Pediatr..

[16] Zhang F. (2026). The Epigenetic-Immune-Microbiome Axis in Early Life: Reprogramming the Origins and Endotypes of Pediatric Asthma. Front. Immunol..

[17] Selma-Royo M., Calatayud Arroyo M., García-Mantrana I., Parra-Llorca A., Escuriet R., Martínez-Costa C., Collado M.C. (2020). Perinatal Environment Shapes Microbiota Colonization and Infant Growth: Impact on Host Response and Intestinal Function. Microbiome.

[18] Galazzo G., Van Best N., Bervoets L., Dapaah I.O., Savelkoul P.H., Hornef M.W., Hutton E.K., Morrison K., Holloway A.C., McDonald H. (2020). Development of the Microbiota and Associations with Birth Mode, Diet, and Atopic Disorders in a Longitudinal Analysis of Stool Samples, Collected from Infancy Through Early Childhood. Gastroenterology.

[19] Dierikx T.H., Visser D.H., Benninga M.A., Van Kaam A.H.L.C., De Boer N.K.H., De Vries R., Van Limbergen J., De Meij T.G.J. (2020). The Influence of Prenatal and Intrapartum Antibiotics on Intestinal Microbiota Colonisation in Infants: A Systematic Review. J. Infect..

[20] Boulund U., Thorsen J., Trivedi U., Tranæs K., Jiang J., Shah S.A., Stokholm J. (2025). The Role of the Early-Life Gut Microbiome in Childhood Asthma. Gut Microbes.

[21] Thänert R., Schwartz D.J., Keen E.C., Hall-Moore C., Wang B., Shaikh N., Ning J., Rouggly-Nickless L.C., Thänert A., Ferreiro A. (2024). Clinical Sequelae of Gut Microbiome Development and Disruption in Hospitalized Preterm Infants. Cell Host Microbe.

[22] Russo D., Lizzi M., Di Filippo P., Di Pillo S., Chiarelli F., Attanasi M. (2022). Time-Specific Factors Influencing the Development of Asthma in Children. Biomedicines.

[23] Kimble R., Gouinguenet P., Ashor A., Stewart C., Deighton K., Matu J., Griffiths A., Malcomson F.C., Joel A., Houghton D. (2023). Effects of a Mediterranean Diet on the Gut Microbiota and Microbial Metabolites: A Systematic Review of Randomized Controlled Trials and Observational Studies. Crit. Rev. Food Sci. Nutr..

[24] Lunjani N., Walsh L.J., Venter C., Power M., MacSharry J., Murphy D.M., O’Mahony L. (2022). Environmental Influences on Childhood Asthma—The Effect of Diet and Microbiome on Asthma. Pediatr. Allergy Immunol..

[25] Temneanu O.R., Mihai A., Olariu R., Oros M., Ioniuc I., Lupu V.V., Lupu A., Grudnicki A., Roșu M.F., Șerban R. (2026). From Diet to Dysbiosis: How Nutritional Factors Shape Gut Microbiota and Drive Airway Inflammation in Pediatric Asthma. Nutrients.

[26] He Z., Wang Z., Yin J., Wang Q. (2025). Research Progress on Microecology and Childhood Respiratory Infections via the Lung-Gut Axis. Front. Pediatr..

[27] Wang Z., Yu J., Liu Y., Gong J., Hu Z., Liu Z. (2025). Role of the Microbiota–Gut–Lung Axis in the Pathogenesis of Pulmonary Disease in Children and Novel Therapeutic Strategies. Front. Immunol..

[28] Mo M., Chen L., Wang Y., Lin X., Li H., Chen B., Yuan J., Tao E. (2026). The Gut-Lung Axis in Childhood Asthma: From Early-Life Programming to Microbiome-Informed Precision Medicine—A Narrative Review. Front. Immunol..

[29] Alcazar C.G.-M., Paes V.M., Shao Y., Oesser C., Miltz A., Lawley T.D., Brocklehurst P., Rodger A., Field N. (2022). The Association between Early-Life Gut Microbiota and Childhood Respiratory Diseases: A Systematic Review. Lancet Microbe.

[30] Valverde-Molina J., García-Marcos L. (2023). Microbiome and Asthma: Microbial Dysbiosis and the Origins, Phenotypes, Persistence, and Severity of Asthma. Nutrients.

[31] Hoskinson C., Petersen C., Turvey S.E. (2025). How the Early Life Microbiome Shapes Immune Programming in Childhood Asthma and Allergies. Mucosal Immunol..

[32] Steininger H., Moltzau-Anderson J., Lynch S.V. (2023). Contributions of the Early-Life Microbiome to Childhood Atopy and Asthma Development. Semin. Immunol..

[33] Aslam R., Herrles L., Aoun R., Pioskowik A., Pietrzyk A. (2024). Link between Gut Microbiota Dysbiosis and Childhood Asthma: Insights from a Systematic Review. J. Allergy Clin. Immunol. Glob..

[34] Van Meel E.R., Attanasi M., Jaddoe V.W.V., Reiss I.K.M., Moll H.A., De Jongste J.C., Duijts L. (2020). *Chlamydia trachomatis* during Pregnancy and Childhood Asthma-Related Morbidity: A Population-Based Prospective Cohort. Eur. Respir. J..

[35] Kloepfer K.M., Kennedy J.L. (2023). Childhood Respiratory Viral Infections and the Microbiome. J. Allergy Clin. Immunol..

[36] Zhu W., Wu Y., Liu H., Jiang C., Huo L. (2021). Gut–Lung Axis: Microbial Crosstalk in Pediatric Respiratory Tract Infections. Front. Immunol..

[37] Casali L., Stella G.M. (2024). The Microbiota in Children and Adolescents with Asthma. Children.

[38] Liu C., Makrinioti H., Saglani S., Bowman M., Lin L.-L., Camargo C.A., Hasegawa K., Zhu Z. (2022). Microbial Dysbiosis and Childhood Asthma Development: Integrated Role of the Airway and Gut Microbiome, Environmental Exposures, and Host Metabolic and Immune Response. Front. Immunol..

[39] Aldriwesh M.G., Al-Mutairi A.M., Alharbi A.S., Aljohani H.Y., Alzahrani N.A., Ajina R., Alanazi A.M. (2023). Paediatric Asthma and the Microbiome: A Systematic Review. Microorganisms.

[40] Russo D., Di Filippo P., Attanasi M., Lizzi M., Di Pillo S., Chiarelli F. (2021). Biologic Therapy and Severe Asthma in Children. Biomedicines.

[41] Hu M., Zhao X., Liu Y., Zhou H., You Y., Xue Z. (2023). Complex Interplay of Gut Microbiota between Obesity and Asthma in Children. Front. Microbiol..

[42] Meng Y., Wang H., Mu D., Zeng S., Wang S. (2026). Host Genetic Variation and Gut Microbiome in Pediatric Diseases. Chin. Med. J..

[43] Zaripova L.N., Midgley A., Christmas S.E., Beresford M.W., Baildam E.M., Oldershaw R.A. (2021). Juvenile Idiopathic Arthritis: From Aetiopathogenesis to Therapeutic Approaches. Pediatr. Rheumatol..

[44] Huang H.Y.R., Wireko A.A., Miteu G.D., Khan A., Roy S., Ferreira T., Garg T., Aji N., Haroon F., Zakariya F. (2024). Advancements and Progress in Juvenile Idiopathic Arthritis: A Review of Pathophysiology and Treatment. Medicine.

[45] Prakken B., Albani S., Martini A. (2011). Juvenile Idiopathic Arthritis. Lancet.

[46] Rigante D., Bosco A., Esposito S. (2015). The Etiology of Juvenile Idiopathic Arthritis. Clin. Rev. Allergy Immunol..

[47] Lee K.-Y., Rhim J.-W. (2026). An Etiopathogenesis of Juvenile Idiopathic Arthritis: The Protein-Homeostasis-System Hypothesis. Front. Pediatr..

[48] La Bella S., Rinaldi M., Di Ludovico A., Di Donato G., Di Donato G., Salpietro V., Chiarelli F., Breda L. (2023). Genetic Background and Molecular Mechanisms of Juvenile Idiopathic Arthritis. Int. J. Mol. Sci..

[49] La Bella S., Alongi A., Filocamo G., Cattalini M., Lanni S., Marino A., Minoia F., Ruperto N. (2026). Environmental Determinants of Juvenile Idiopathic Arthritis: Current Evidence and Future Directions. Glob. Pediatr..

[50] Ricciuto A., Sherman P.M., Laxer R.M. (2020). Gut Microbiota in Chronic Inflammatory Disorders: A Focus on Pediatric Inflammatory Bowel Diseases and Juvenile Idiopathic Arthritis. Clin. Immunol..

[51] Pelanda H., Rulli E., Sultanov M., Adornato S., Rigante D. (2026). Highlights on the Contribution of Gut Microbiota to Immune-Mediated Diseases in Childhood. Mediterr. J. Hematol. Infect. Dis..

[52] Romero-Figueroa M.D.S., Ramírez-Durán N., Montiel-Jarquín A.J., Horta-Baas G. (2023). Gut-Joint Axis: Gut Dysbiosis Can Contribute to the Onset of Rheumatoid Arthritis via Multiple Pathways. Front. Cell. Infect. Microbiol..

[53] Petty R.E., Southwood T.R., Manners P., Baum J., Glass D.N., Goldenberg J., He X., Maldonado-Cocco J., Orozco-Alcala J., Prieur A.-M. (2004). International League of Associations for Rheumatology Classification of Juvenile Idiopathic Arthritis: Second Revision, Edmonton, 2001. J. Rheumatol..

[54] Srinivasalu H., Treemarcki E.B., Redmond C. (2021). Advances in Juvenile Spondyloarthritis. Curr. Rheumatol. Rep..

[55] Qian X., Liu Y.-X., Ye X., Zheng W., Lv S., Mo M., Lin J., Wang W., Wang W., Zhang X. (2020). Gut Microbiota in Children with Juvenile Idiopathic Arthritis: Characteristics, Biomarker Identification, and Usefulness in Clinical Prediction. BMC Genom..

[56] Arvonen M., Vänni P., Sarangi A.N., V Tejesvi M., Vähäsalo P., Aggarwal A., Stoll M.L. (2020). Microbial Orchestra in Juvenile Idiopathic Arthritis: Sounds of Disarray?. Immunol. Rev..

[57] Wang Y., Wei J., Zhang W., Doherty M., Zhang Y., Xie H., Li W., Wang N., Lei G., Zeng C. (2022). Gut Dysbiosis in Rheumatic Diseases: A Systematic Review and Meta-Analysis of 92 Observational Studies. eBioMedicine.

[58] Cole L.D., Kuhn K.A. (2025). It Takes a Village. Rheum. Dis. Clin. N. Am..

[59] Harjacek M. (2021). Immunopathophysiology of Juvenile Spondyloarthritis (jSpA): The “Out of the Box” View on Epigenetics, Neuroendocrine Pathways and Role of the Macrophage Migration Inhibitory Factor (MIF). Front. Med..

[60] Lee Y.-S., Stetson R.C., Madrigal J.M., Orandi A.B., Hollender H., Gor K., George R.E., Sprong M.E. (2026). Association between Asthma and Juvenile Idiopathic Arthritis in Children in the United States: A Propensity Score Weighted Cross-Sectional Study. Pediatr. Rheumatol..

[61] Wu H.-J., Ivanov I.I., Darce J., Hattori K., Shima T., Umesaki Y., Littman D.R., Benoist C., Mathis D. (2010). Gut-Residing Segmented Filamentous Bacteria Drive Autoimmune Arthritis via T Helper 17 Cells. Immunity.

[62] Teng F., Klinger C.N., Felix K.M., Bradley C.P., Wu E., Tran N.L., Umesaki Y., Wu H.-J.J. (2016). Gut Microbiota Drive Autoimmune Arthritis by Promoting Differentiation and Migration of Peyer’s Patch T Follicular Helper Cells. Immunity.

[63] Fan T., Tai C., Sleiman K.C., Cutcliffe M.P., Kim H., Liu Y., Li J., Xin G., Grashel M., Baert L. (2025). Aberrant T Follicular Helper Cells Generated by TH17 Cell Plasticity in the Gut Promote Extraintestinal Autoimmunity. Nat. Immunol..

[64] Bradley C.P., Teng F., Felix K.M., Sano T., Naskar D., Block K.E., Huang H., Knox K.S., Littman D.R., Wu H.-J.J. (2017). Segmented Filamentous Bacteria Provoke Lung Autoimmunity by Inducing Gut-Lung Axis Th17 Cells Expressing Dual TCRs. Cell Host Microbe.

[65] Nistala K., Moncrieffe H., Newton K.R., Varsani H., Hunter P., Wedderburn L.R. (2008). Interleukin-17–Producing T Cells Are Enriched in the Joints of Children with Arthritis, but Have a Reciprocal Relationship to Regulatory T Cell Numbers. Arthritis Rheum..

[66] Pianta A., Arvikar S.L., Strle K., Drouin E.E., Wang Q., Costello C.E., Steere A.C. (2017). Two Rheumatoid Arthritis–Specific Autoantigens Correlate Microbial Immunity with Autoimmune Responses in Joints. J. Clin. Investig..

[67] Wang Y.-H., Yan Z.-Z., Luo S.-D., Hu J.-J., Wu M., Zhao J., Liu W.-F., Li C., Liu K.-X. (2023). Gut Microbiota-Derived Succinate Aggravates Acute Lung Injury after Intestinal Ischaemia/Reperfusion in Mice. Eur. Respir. J..

[68] Littlewood-Evans A., Sarret S., Apfel V., Loesle P., Dawson J., Zhang J., Muller A., Tigani B., Kneuer R., Patel S. (2016). GPR91 Senses Extracellular Succinate Released from Inflammatory Macrophages and Exacerbates Rheumatoid Arthritis. J. Exp. Med..

[69] Venken K., Jarlborg M., Stevenaert F., Malfait T.L.A., Vlieghe C., Abraham Y., Manuello T., Decruy T., Vanhee S., Wils H. (2025). Shared Lung and Joint T Cell Repertoire in Early Rheumatoid Arthritis Driven by Cigarette Smoking. Ann. Rheum. Dis..

[70] Rossetti M., Spreafico R., Consolaro A., Leong J.Y., Chua C., Massa M., Saidin S., Magni-Manzoni S., Arkachaisri T., Wallace C.A. (2017). TCR Repertoire Sequencing Identifies Synovial Treg Cell Clonotypes in the Bloodstream during Active Inflammation in Human Arthritis. Ann. Rheum. Dis..

[71] Dick S., Turner S. (2020). The Airway Microbiome and Childhood Asthma—What Is the Link?. Acta Med. Acad..

[72] Lupu A., Jechel E., Mihai C.M., Mitrofan E.C., Fotea S., Starcea I.M., Ioniuc I., Mocanu A., Ghica D.C., Popp A. (2023). The Footprint of Microbiome in Pediatric Asthma—A Complex Puzzle for a Balanced Development. Nutrients.

[73] Ma Y., Zheng H., Tian H., Cui J., Liu L. (2025). Research Advance in Correlation between Childhood Asthma and Gut Microbiota. Front. Cell. Infect. Microbiol..

[74] Zhang L., Wu L., Shi C., Huang Q., Zhan L., Zeng Y., Deng Q. (2026). The Role of Intestinal Microbiota in the Pathogenesis of Childhood Asthma. Front. Immunol..

