# Supplementary material for: The Gut–Lung–Joint Axis: A Conceptual Framework for Microbiome-Mediated Crosstalk Between Respiratory and Rheumatic Diseases in Childhood
Source: Int J Mol Sci. 2026 Sep 29;27(19):8692. doi: 10.3390/ijms27198692 (PMC13664819; doi:10.3390/ijms27198692)
Supplement: Supplementary file 1 [file ijms-27-08692-s001.zip › ijms-4534891-supplementary.pdf]

Table S1. Reviews included in the gut–lung axis evidence synthesis.

Study	Review design	Population scope	Microbiome scope	Main focus	Diseases or clinical outcomes
Dick and Turner, 2020 [71]	Review	Pediatric	Gut–airway	Airway and gut microbiome in asthma	Childhood asthma
Zhu et al., 2021 [36]	Narrative review	Pediatric	Gut–lung axis	gut–lung microbiota crosstalk	Respiratory infections
García-Mauriño Alcázar et al., 2022 [29]	Systematic review	Pediatric	Gut/intestinal	Early-life gut microbiota	Respiratory infections, recurrent wheezing, and asthma
Stricker et al., 2022 [9]	Narrative review	Pediatric	Gut–airway	Intestinal and respiratory microbiota	Pediatric pulmonary diseases
Liu et al., 2022 [38]	Review	Pediatric	Gut–airway	Dysbiosis, environmental exposures, metabolic, immune mechanisms	Childhood asthma
Lunjani et al., 2022 [24]	Review	Pediatric	Gut/intestinal	Diet-microbiome interactions	Childhood asthma
Aldriwesh et al., 2023 [39]	Systematic review	Pediatric	Gut–airway	Airway and gut microbiome	Pediatric asthma
Hu et al., 2023 [41]	Narrative review	Pediatric	Gut/intestinal	Gut microbiota as a mediator in obesity-asthma	Obesity-related childhood asthma
Kloepfer and Kennedy, 2023 [35]	Narrative review	Pediatric	Gut–airway	Respiratory viral infections and gut microbiota	Wheezing/ asthma development/exacerbations
Lupu et al., 2023 [72]	Narrative review	Pediatric	Gut–airway	Intestinal and pulmonary microbiome dysbiosis	Pediatric asthma
Steininger et al., 2023 [32]	Review	Pediatric	Gut–airway	Early-life microbiome and immune development	Childhood atopy and asthma
Valverde-Molina and Garcia-Marcos, 2023 [30]	Narrative review	Pediatric	Gut–airway	Gut and airway microbial dysbiosis	Asthma severity
Aslam et al., 2024 [33]	Systematic review	Pediatric	Gut/intestinal	Gut microbiota composition during infancy and childhood	Atopy and asthma in childhood
Casali and Stella, 2024 [37]	Narrative review	Pediatric	Gut–airway	Gut and respiratory microbiota in asthma	Childhood and adolescent asthma
Boulund et al., 2025 [20]	Review	Pediatric	Gut/intestinal	Early-life microbiota	Childhood asthma
He et al., 2025 [26]	Narrative review	Pediatric	Gut–lung axis	Microbial ecology and the gut–lung axis	Respiratory infections
Hoskinson et al., 2025 [31]	Review	Pediatric	Gut–intestinal	Gut microbiome maturation and immune programming	Childhood asthma
Liu et al., 2025 [10]	Narrative review	Pediatric	Gut–airway	Development and composition of the respiratory and intestinal microbiota	Pediatric asthma phenotypes and endotypes

Ma et al., 2025 [73]	Review	Pediatric	Gut–lung axis	Gut microbiota and immune modulation	Childhood asthma
Wang et al., 2025 [27]	Narrative review	Pediatric	Gut–lung axis	Microbiota–gut–lung axis	Pediatric asthma
Meng et al., 2026 [42]	Narrative review	Pediatric	Gut/intestinal	Interactions between host genetic variation and the gut microbiome	Childhood asthma
Mo et al., 2026 [28]	Narrative review	Pediatric	Gut–lung axis	Early-life microbial programming	Childhood asthma
Temneanu et al., 2026 [25]	Narrative review	Pediatric	Gut–lung axis	Diet, gut dysbiosis, and airway inflammation	Pediatric asthma, including obesity related asthma
Wang et al., 2026 [15]	Review	Pediatric	Gut/intestinal	Early-life microbiota and immune programming	Childhood asthma prevention
Zhang F., 2026 [16]	Review	Pediatric	Gut–lung axis	Epigenetic-immune-microbiome interactions during early life	Endotypes of pediatric asthma
Zhang L. et al., 2026 [74]	Review	Pediatric	Gut–lung axis	Intestinal microbiome and immune mechanisms	Childhood asthma pathogenesis

Table S2. Reviews included in the gut–joint axis evidence synthesis.

Study	Review design	Population scope	Microbiome scope	Main focus	Diseases or clinical outcomes
Arvonen et al., 2020 [56]	Narrative review	Pediatric	Gut/intestinal	Early-life factors, gut microbiota, immune mechanisms	JIA
Ricciuto et al., 2020 [50]	Narrative review	Mixed	Gut/intestinal	Th17/Treg regulation and microbiota target interventions	Pediatric IBD and JIA/inflammatory arthropathies
Harjacek, 2021 [59]	Narrative review	Pediatric	Gut–joint axis	Gut dysbiosis, IL-17/IL-23 pathways, epigenetic and innate immune mechanisms in juvenile spondyloarthritis	Juvenile spondyloarthritis, including enthesitis-related arthritis
Xin et al., 2021 [7]	Narrative review	Pediatric	Gut/intestinal	Intestinal microbiota and environmental exposures	JIA
Srinivasalu et al., 2021 [54]	Narrative review	Pediatric	Gut–joint axis	Pathogenesis, gut microbiome	Juvenile spondyloarthritis, including enthesitis-related arthritis
Wang et al., 2022 [57]	Systematic review	Mixed	Gut/intestinal	Gut microbial diversity and composition	Rheumatic diseases, including JIA
Cole and Kuhn, 2025 [58]	Narrative review	Pediatric	Oral and gut/intestinal	Microbiome-related early-life risk factors, oral and fecal dysbiosis	JIA
La Bella et al., 2026 [49]	Narrative review	Pediatric	Gut/intestinal	Environmental and early-life determinants of JIA	JIA
Matei and Rosser, 2026 [6]	Narrative review	Pediatric	Gut–joint axis	Gut–joint axis, perinatal factors, immune mechanisms	JIA and enthesitis-related arthritis
Pelanda et al., 2026 [51]	Narrative review	Pediatric	Gut/intestinal	Gut microbiota and immune regulation	JIA and other pediatric immune-mediated inflammatory diseases
Lee and Rhim, 2026 [47]	Narrative review	Pediatric	Gut/intestinal	Microbiota-related triggers, immune activation	JIA

Abbreviations: IBD, inflammatory bowel disease; IL, interleukin; JIA, juvenile idiopathic arthritis; Th17, T helper 17; Treg, regulatory T cell.
